# UCEasy: A software package for automating and simplifying the analysis of ultraconserved elements (UCEs)

**DOI:** 10.3897/BDJ.9.e78132

**Published:** 2021-12-10

**Authors:** Caio V. R. Ribeiro, Lucas P. Oliveira, Romina Batista, Marcos De Sousa

**Affiliations:** 1 Coordenação de Ciência da Computação, Centro Universitário do Estado do Pará (CESUPA), Belém, Brazil Coordenação de Ciência da Computação, Centro Universitário do Estado do Pará (CESUPA) Belém Brazil; 2 Instituto de Computação, Universidade Estadual de Campinas (UNICAMP), Campinas, Brazil Instituto de Computação, Universidade Estadual de Campinas (UNICAMP) Campinas Brazil; 3 Instituto Nacional de Pesquisas da Amazônia (INPA), Manaus, Brazil Instituto Nacional de Pesquisas da Amazônia (INPA) Manaus Brazil; 4 Gothenburg Global Biodiversity Centre, Gothenburg, Sweden Gothenburg Global Biodiversity Centre Gothenburg Sweden; 5 Museu Paraense Emílio Goeldi (MPEG), Belém, Brazil Museu Paraense Emílio Goeldi (MPEG) Belém Brazil

**Keywords:** phylogenomics, ultraconserved elements (UCEs), research software, reproducibility, bioinformatics.

## Abstract

**Background:**

The use of Ultraconserved Elements (UCEs) as genetic markers in phylogenomics has become popular and has provided promising results. Although UCE data can be easily obtained from targeted enriched sequencing, the protocol for *in silico* analysis of UCEs consist of the execution of heterogeneous and complex tools, a challenge for scientists without training in bioinformatics. Developing tools with the adoption of best practices in research software can lessen this problem by improving the execution of computational experiments, thus promoting better reproducibility.

**New information:**

We present UCEasy, an easy-to-install and easy-to-use software package with a simple command line interface that facilitates the computational analysis of UCEs from sequencing samples, following the best practices of research software. UCEasy is a wrapper that standardises, automates and simplifies the quality control of raw reads, assembly and extraction and alignment of UCEs, generating at the end a data matrix with different levels of completeness that can be used to infer phylogenetic trees. We demonstrate the functionalities of UCEasy by reproducing the published results of phylogenomic studies of the bird genus *Turdus* (Aves) and of Adephaga families (Coleoptera) containing genomic datasets to efficiently extract UCEs.

## Introduction

In the last decade, new genome-subsampling methods have been developed as a cheaper and simpler alternative to complete genome sequencing, thus enabling the scientific community to better understand the evolutionary inter-relationships of species (Davey et al. 2011, Andermann et al. 2020). One of these methods concentrates the sequencing effort on sets of pre-selected genetic markers, a reduced set of the genome. Ultraconserved Elements (UCEs) are amongst the most commonly used capture baits to target highly-conserved regions spread across a genome. These genomic regions are strongly conserved in different species throughout multiple evolutionary timescales (Faircloth et al. 2012) and have been efficiently used as molecular markers for phylogenomic studies (Bejerano et al. 2004, Baca et al. 2017, Branstetter et al. 2017).

Although UCE data can be easily obtained from targeted enriched sequencing (McCormack et al. 2011, Faircloth et al. 2012), the protocols for *in silico* analysis of UCEs consists of the execution of many heterogeneous and complex tools. Currently, the most widely used bioinformatics pipeline for processing UCE data is the software package PHYLUCE (Faircloth 2015). Amongst the main tasks performed by this software package are: quality control, assembly and extraction and alignment of UCEs for downstream analysis. Other phylogenomic tools use the PHYLUCE pipeline, such as seqcap_pop (Harvey et al. 2016) to obtain phased alignments of UCEs and MitoFinder (Allio et al. 2020) to extract UCE and mitogenomic data. Although the PHYLUCE pipeline is robust and well-established, it requires the execution of many command line scripts from heterogeneous tools, which can be quite challenging for scientists without sufficient training in bioinformatics.

Magee et al. (2014) showed that 60% of published phylogenetic analyses are not reproducible. Many types of software used in bioinformatics pipelines have been developed without the adoption of best practices in research software (Jiménez et al. 2017). Furthermore, making data and code available does not guarantee reproducibility (Beaulieu-Jones and Greene 2017, Ferenhof and Alves de Sousa 2021). A bioinformatics tool that adheres to recommendations for best practices in research software offers a greater guarantee of computational reproducibility (Sandve et al. 2013, Wilson et al. 2014, Leprevost et al. 2014, Piccolo and Frampton 2016, Jiménez et al. 2017). The main recommendations are: i) Use package managers in order to make installation easier and to ensure that software versions and dependencies are installed correctly. Difficult-to-install software can be frustrating, impact reliability and impair reproducibility (Georgeson et al. 2019); ii) Make your software source code and documentation available in public and indexed repositories through DOI as a way to promote accessibility; iii) Adopt an OSS (Open Source Software) licence, since unlicensed software discourages reusability and collaboration (Georgeson et al. 2019). The use of OSS licences improves accessibility, reusability and transparency and contributes to the reproducibility of the results generated by the software (Jiménez et al. 2017); iv) Use a version control system to maintain the history of changes made to the source code, allowing arbitrary versions to be retrieved and compared, thus providing provenance for the code; v) When it comes to command-line interface (CLI) tools, implement commands with consistent, distinct and meaningful names, besides clear output and error messages (Seemann 2013, Wilson et al. 2014); vi) When a bioinformatics analysis uses a pipeline containing heterogeneous tools, recording the execution progress of each tool in a log file is good practice. The metadata obtained in this manner should contain the commands executed, the generated output and the date and time of occurrence, all of which provide useful information to aid in debugging and provenance of the pipeline execution (Georgeson et al. 2019).

In this work, we present UCEasy, an open source software package that facilitates the analysis of UCEs from sequencing samples, following the best practices of research software. UCEasy is a Python wrapper that standardises, automates and simplifies the following PHYLUCE tasks: quality control of raw reads, assembly, alignment and UCE extraction. We demonstrate the functionalities of UCEasy by reproducing the published results from two phylogenomic studies (Baca et al. 2017, Batista et al. 2020) to efficiently extract UCEs.

## Project description

### Title

UCEasy

### Design description

**Figure 1.**
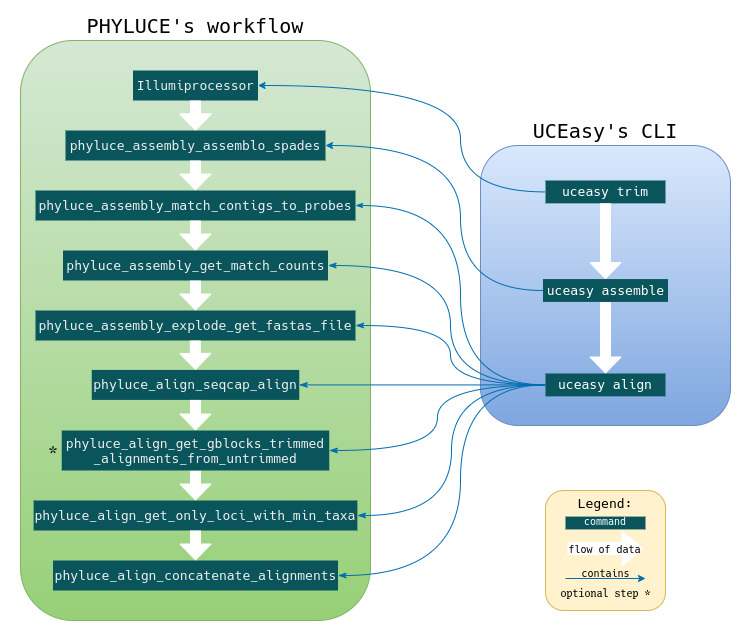
UCEasy's CLIs interacting with PHYLUCE's workflow.

UCEasy automates and simplifies the analysis of UCE datasets from DNA sequence samples in FASTQ files (either single-ended or paired-ended), interacting with Python scripts adopted in the standard PHYLUCE 1.6 workflow (https://phyluce.readthedocs.io/en/latest/tutorials/tutorial-1.html), as shown in Fig. 1. The UCEasy CLI is organised into three modules: i) Trim: this module is responsible for quality control, removing adapters and low quality reads using the command **uceasy trim**. The user needs to specify the directory that contains the raw sequence files (.fastq) and also the CSV file that contains the barcode of the samples and sequence adapters; ii) Assemble: this module receives the clean reads by the Quality Control and then uses SPAdes (Bankevich et al. 2012) to perform the assembly of the contigs without the use of a reference genome using the command **uceasy assemble**; iii) Align: in this module, the extraction and alignment of UCEs is performed with the command **uceasy align**. The contigs matching UCE with a pre-selected UCE probe-set (https://github.com/faircloth-lab/uce-probe-sets) are identified to create a list of UCEs by sample. This list of UCEs is used to extract UCE contigs from de novo assemblies on a sample-by-sample basis, generating several FASTA files. Then, the data are aligned against all these FASTA files using MAFFT (Katoh and Standley 2013) or MUSCLE (Edgar 2004). The next step is the generation of alignment matrices with different levels of completeness: either all taxa may have data for all UCEs or some UCEs are presented for a certain percentage of taxa. Finally, these data matrices are concatenated to either NEXUS or PHYLIP file format, that can be used to infer phylogenetic trees using programmes such as RAxML or ExaBayes. More details about the UCEasy commands and their parameters, as well as a comparison between CLIs demonstrating the ease of use of UCEasy compared to PHYLUCE, can be found on our wiki (https://github.com/uceasy/uceasy/wiki).

## Web location (URIs)

Homepage: https://github.com/uceasy/uceasy

Wiki: https://github.com/uceasy/uceasy/wiki

## Technical specification

Platform: Linux

Programming language: Python 3.7

Operational system: GNU/Linux; Hardware requirements (Minimum): 16 GB of RAM, 8 core CPU

## Repository

Type: Github

Browse URI: https://github.com/uceasy/uceasy

## Usage licence

### Usage licence

Other

### IP rights notes

MIT Licence

## Implementation

### Implements specification

**Figure 2.**
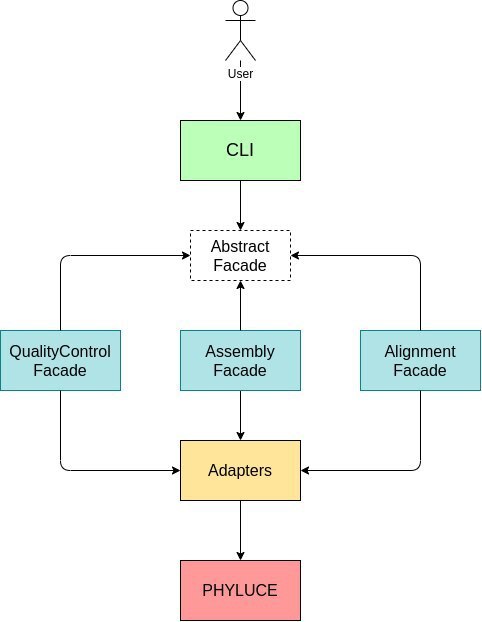
UCEasy software architecture. The coloured boxes represent components and arrows the dependencies between them. The Abstract Facade implements a common interface with which the other facades have to comply.

UCEasy has an extensible software architecture that makes use of the Facade and Adapter design patterns (Gamma et al. 1994). The Facade pattern aims to provide a unified interface to a set of components in a subsystem, improving its usability. The idea is to add new modules in the future, as different types of UCE analysis emerge. The Adapter pattern is used when a new package needs to be integrated with an existing system, but the new package and the system have different structures without a direct interface. We applied this pattern to integrate UCEasy with PHYLUCE. The Facade and Adapter patterns allow the interaction between UCEasy and PHYLUCE without the need to change the source code of PHYLUCE. The UCEasy software architecture is shown in Fig. 2.

UCEasy was built based on best practices in scientific computing (Wilson et al. 2014) by adopting the following recommendations: a) Package manager: UCEasy uses pip (https://pip.pypa.io) as the package manager, easing the installation process. The UCEasy install command: pip install uceasy; b) Public software repositories: the source code and documentation of UCEasy is available in the public repositories of PyPI (https://pypi.org/project/uceasy) and Github (https://github.com/uceasy/uceasy). In addition, the package is indexed in the Zenodo repository with DOI: 10.5281/zenodo.5225152, in order to make it discoverable, searchable and referable to as many communities as possible; c) Software licence: UCEasy adopts the MIT licence (https://opensource.org/licenses/MIT), which gives full freedom of use, copying, modification and distribution of the source code, thereby enabling reuse and scientific collaboration; d) Version control system: the UCEasy source code and documentation are kept publicly on Github, a repository widely used by the scientific community (Perez-Riverol et al. 2016), allowing other developers to follow and contribute to the evolution of UCEasy; e) CLI standard: UCEasy follows main recommendations for making command lines usable (Seemann 2013, Georgeson et al. 2019), keeping commands associable, consistent and memorable. UCEasy commands adopt the POSIX standard as a way to help the user (Zlotnick 1991); f) UCEasy offers an optional progress-logging facility, using the uceasy --tracking-file (or -t) command. The log file contains the command line used to run the programme, the output generated (e.g. errors, warnings and messages) and the date and time the execution started and ended.

### Audience

The target audience for this software package includes evolutionary biologists and conservation scientists with knowledge of basic Linux commands. We are open to discussing additional ideas or new features to expand the current functionality of this software package.

## Additional information

### Results and conclusion

To demonstrate the effectiveness of UCEasy, we reproduced the published results by Baca et al. (2017) and Batista et al. (2020) to extract UCE for downstream phylogenomics analyses. We ran UCEasy on an Ubuntu 20.04 server with 32-core CPU and 64 GB of RAM to compare the results.

Baca et al. (2017) used UCEs to reconstruct the phylogeny of Adephaga families (Coleoptera), which had proven to be a major challenge because of their exceptional species richness, complicated morphological characteristics and sparse molecular data. Baca et al. (2017) used a bait set to target 5k UCEs (Faircloth 2017). A genomic dataset containing 20 raw sequence reads (with a data volume of 8 Gbases and 3,407 Mbytes), available under BioProject accession PRJNA379181, was downloaded and processed.

The study of Batista et al. (2020) sequenced genomic data in order to solve controversies surrounding the early diversification and biogeography of the genus *Turdus* (Aves, Turdidae). Batista et al. (2020) used a bait set to target 2.5k UCEs (Faircloth et al. 2012), as well as specific probes for *Turdus*, based on 49 of the genetic markers described in Backström et al. (2007). We downloaded and processed a genomic dataset of 115 raw sequence reads (with a data volume of 37 Gbases and 23,333 Mbytes) used in Batista et al. (2020) available at NCBI under the BioProject accession PRJNA574741.

We captured significantly more UCEs than Baca et al. 2017 (305 at 50% vs. 334 at 50% in this study) and Batista et al. (2020) (2,312 of total UCEs, 854 at 85%, 1,931 at 75% vs. 2,436 of total UCEs, 1,708 at 85%, 2,244 at 75% in this study). The study by Baca et al. (2017) was reproduced in a total time of 16 hours and 15 minutes and by Batista et al. (2020) in 63 hours and 47 minutes. In both published studies, the Trinity assembler was used. UCEasy used Spades which gave us better results.

UCEasy successfully reproduced the pipeline of the studies mentioned and met the best practices recommended in the literature for scientific computing. A standardised package, such as that presented here, can help evolutionary biologists by automating laborious tasks and facilitating the reproducibility of computational experiments. Finally, UCEasy architecture is sufficiently robust to support new updates from PHYLUCE without hassle. As future work, we plan to extend UCEasy to include the new PHYLUCE 1.7 version and incorporate new phylogenetic software packages from other developers.
